# Identification of the key immune-related genes in aneurysmal subarachnoid hemorrhage

**DOI:** 10.3389/fnmol.2022.931753

**Published:** 2022-09-12

**Authors:** Xing Wang, Dingke Wen, Chao You, Lu Ma

**Affiliations:** ^1^Department of Neurosurgery, West China Hospital, Sichuan University, Chengdu, China; ^2^West China Brain Research Centre, Sichuan University, Chengdu, China

**Keywords:** subarachnoid hemorrhage, intracranial aneurysm, inflammatory activation, neutrophils, immune cells

## Abstract

Subarachnoid hemorrhage (SAH) is a major cause of death and morbidity worldwide, often due to rupture of intracranial aneurysms (IAs). Immune infiltration and inflammatory activation play key roles in the process of aneurysmal SAH (aSAH). This study aimed to elaborate the immune infiltration and identify related biomarkers both in blood and tissue samples from patients with aSAH. Expression data of aSAH and healthy control samples were obtained from gene expression omnibus (GEO) database. Overall, a blood sample dataset GSE36791 and a tissue sample dataset GSE122897 were included. Differentially expressed genes (DEGs) between aSAH and healthy samples were explored. We applied GO biological and Gene Set Enrichment Analyses (GSEA) processes to access the functional enrichment. Then feature elimination algorithms based on random forest were used to screen and verify the biomarkers of aSAH. We performed three computational algorithms including Cell type Identification by Estimating Relative Subsets of RNA Transcripts (CIBERSORT), Microenvironment Cell Populations-counter (MCPcounter), and xcell to evaluate the immune cell infiltration landscape to identify the unique infiltration characteristics associated with rupturing. We found 2,220 DEGs (856 upregulated and 1,364 downregulated) in the original dataset. Functional analysis revealed most of these genes are enriched in immunological process, especially related with neutrophil response. Similar signaling pathway enrichment patterns were observed in tissue sample dataset and ClueGo. Analysis of immune microenvironment infiltration suggested neutrophils were abnormally upregulated in aSAH compared with those in the control group. Key gene SRPK1 was then filtered based on feature elimination algorithms, and transcription factor (TF) ZNF281 is assumed to participate in immunomodulation by regulating expression of SRPK1. Several immunomodulators such as CXCR1 and CXCR2 also appear to be involved in the progression of aSAH. In the present study, we performed a comprehensive stratification and quantification of the immune infiltration status of aSAH. By exploring the potential mechanism for aSAH based on several computational algorithms, key genes including SRPK1 and ZNF281 were filtered. This study may be of benefit to patients who are at high risk of suffering aSAH which allows for early diagnosis and potential therapy.

## Introduction

It is estimated that 3% of the population live with unruptured intracranial aneurysms (IA; Vlak et al., 2011). Rupture of an intracranial aneurysm leads to aneurysmal subarachnoid hemorrhage (aSAH), a devastating cerebrovascular disease. Despite developments in medical care and surgical treatment, the case fatality rate due to aSAH remains high. According to the publications, the mortality rate is estimated to be up to 66.7% (Macdonald and Schweizer, 2017). Even if patients survive the initial bleeding, they are at high risk of being left with disabilities and functional impairments. It reported the permanent disability rates was approximately 50% (van Gijn et al., 2007). Of particular attention is the much younger age of patients affected by aSAH compared to ischemic stroke and the resultant higher socio-economic burden (van Gijn et al., 2007; Macdonald and Schweizer, 2017). Therefore, it is important to understand the cellular and molecular mechanisms involved in the progression and rupture of intracranial aneurysms.

Numerous factors appear to contribute to early and late brain injury after aSAH (Fujii et al., 2013). However, immune reactions following SAH have been recognized as a pivotal determinant in secondary brain injury (Prunell et al., 2005; Schneider et al., 2018; Suzuki, 2019). Following rupture of the aneurysm, components of the blood including broken red blood cell debris, cytokines, and immune cells enter the brain tissue and are key initiators of the inflammatory cascade response (Muhammad and Hänggi, 2021). This pernicious cycle of activity may lead to almost all processes involved in aSAH, including apoptotic or necrotic cell death, cerebral vasospasm, delayed cerebral ischemia, hydrocephalus, and multiple organ infections (Heinz et al., 2021). Recently, emerging evidence suggest that neutrophil-induced inflammation play a specific role among these immune reactions (Tutino et al., 2018a, b). For example, observational studies over the past four decades reported an association between early elevated neutrophil counts after the onset of aSAH and undesirable outcomes, including hydrocephalus, vasospasm, diminished neurological function, and death (Provencio et al., 2010; Chou et al., 2011; Zhang B. et al., 2021; Zhang Y. et al., 2021).

It is important to note that most regular blood work-up biomarkers remain largely the same between healthy individuals and patients with unruptured aneurysms. Only recent studies reported circulatory long non-coding RNAs or miRNAs that might be of diagnostic values, however the prediction accuracy of these models remain to be validated in external cohorts (Poppenberg et al., 2020; Tutino et al., 2020). In terms of mechanism, aSAH could be triggered by occult and sudden pathophysiological changes at histological or molecular level, regardless of the confirmed preceding aneurysm development. This may commonly be observed in cases like arterial dissecting aneurysms or blister-like aneurysms. Moreover, many studies have implied most culprit subarachnoid hemorrhage have minor sentinel bleeding that might alter the microenvironment of arterial condition. Hence gaining a deeper understanding between otherwise healthy individuals and aneurysm rupture patients might be a promising study strategy to consider.

To detect a circulatory predictor that may be indicative of imminent aneurysmal hemorrhage and to further elucidate the underlying mechanisms associated with IA progression and rupture, we conducted this study and concentrated on the cellular and molecular features involved in the progression of aSAH. We aimed to identify novel molecules that are involved in both blood and tissue inflammatory responses, which might be beneficial for the timely diagnosis and identification of potential therapeutic targets for this fatal disease.

## Materials and Methods

### Data collection and expression analysis

Publicly available transcriptome data of blood samples from aSAH patients and healthy participates with available clinical information were systematically reviewed from the Gene Expression Omnibus (GEO) database. One microarray dataset named GSE36791 was included in the study. This dataset contained 43 whole blood samples from aSAH patients and 18 healthy blood samples. Another RNA-sequencing (RNAseq) dataset named GSE122897 was also included. This dataset contained 22 ruptured aneurysmal tissues and 16 healthy arterial samples. All the RNAseq and microarray data were normalized and log2 transformed before analysis. Besides, 24 pairs of artery tissue samples and 12 pairs of blood samples from aSAH patients and healthy controls were obtained from our hospital.

### Western-blotting analysis

Human blood samples and artery tissue samples were collected for western blot. Briefly, an equal amount of protein from each sample was prepared and separated by an 10% sodium dodecyl sulfate polyacrylamide, and transferred onto polyvinylidene fluoride (PVDF) membranes. After that, the membranes were blocked with skim milk buffer for 2 h, and then incubated at 4°C overnight with the following antibodies: anti-SRPK1 (1:1,000 dilution, A12510, ABclonal, China), anti-ZNF281 (1:2,000 dilution, A12650, ABclonal, China), anti-S100A8 (1:1,000 dilution, A15315, ABclonal, China), anti-S100A9 (1:1,000 dilution, A9842, ABclonal, China), and anti-GAPDH (1:5,000 dilution, AC001, ABclonal, China). After that, the PVDF membrane was incubated with a secondary antibody (goat anti-rabbit antibody, 1:5,000 dilution, AS014, Abclonal, China) for 2 h, and then washed with TBST three times. The ECL detection kit (Epizyme biotech, China) was used to visualize protein band. Images were analyzed by Image J software (Image J 1.53, NIH, USA).

### Immunofluorescence staining

Briefly, frozen artery tissues were sliced into 4–6 μm sections. The slides were then blocked with 5% BSA for 1 h. After that, primary antibodies against SRPK1 (1:100 dilution, A12510, ABclonal, China), ZNF281 (1:100 dilution, A12650, ABclonal, China), S100A8 (1:100 dilution, A15315, ABclonal, China), and S100A9 (1:100 dilution, A9842, ABclonal, China) were incubated overnight at 4°C. After washing with TBS, the cryosections were incubated for 1 h with the secondary antibody (goat anti-rabbit antibody, 1:100 dilution, AS011, ABclonal, China). The sections were visualized using a fluorescence microscope. Images were analyzed by Image J software (Image J 1.53, NIH, USA).

### Identification of differentially expressed genes (DEGs)

R packages “limma” and “DESeq2” were used to obtain differentially expressed genes (DEGs) for microarray data and RNA-seq reads count data, respectively. The genes with an absolute value of log2(foldchange) larger than 0.25 and adjusted p-value less than 0.05 were considered as specific genes. The R packages “pheatmap” and “ggplot2” were applied to present results as heatmaps and volcano plots.

### Gene ontology (GO) analysis and reactome enrichment analysis

GO analysis on the aberrantly expressed genes between aSAH and healthy control samples was performed based on the Database for Annotation, Visualization and Integrated Discovery (DAVID) database (Dennis et al., 2003). The list of gene IDs, as well as their log2(foldchange) values was used as the input file. GO terms with an adjusted *p*-value less than 0.05 were considered statistically significant. The reactome enrichment analysis was performed with the enrichPathway function in the “ReactomePA” package.

### Random forest and receiver operating characteristic (ROC)

We used a random forest algorithm, which is based on a multitude of decision trees. This method was applied to filter the most important candidates associated with different features. Candidate genes were used as the input of random forest to construct prediction models. This procedure was applied using the R package “randomForest” with 2,000 trees. The aim was to identify the top 15 key genes.

ROC and the area under the curve (AUC) value were then performed to compare the predictive sensitivity and specificity of concerned genes in differentiating between aSAH samples and control samples. These analyses were derived using the “pROC” package.

### Gene set variation analysis (GSVA)

GSVA enrichment was performed by the R package “GSVA” using the heatmap function. This analysis was applied to perform immunologic signature of GSEA. Using the “limma” package, an adjusted *p*-value less than 0.05 with false discovery rate (FDR) <0.05 was considered to represent statistical significance between groups. Related datasets were retrieved from Molecular Signatures Database (MSigDB; Liberzon et al., 2015).

### Evaluation of immune cell infiltration

The landscape of immune cell infiltration was evaluated by three algorithms, named Cell type Identification by Estimating Relative Subsets of RNA Transcripts (CIBERSORT), Microenvironment Cell Populations-counter (MCPcounter), and xcell. All the methods were used to quantify the relative or absolute abundance of immune cell populations in different samples.

CIBERSORT algorithm provides an estimation of the abundance of 22 human hematopoietic cell phenotypes using a leukocyte gene signature matrix of 547 genes (Newman et al., 2015). The analysis was performed using the CIBERSORT source code. The abundance score of each immune cell population was generated through the “MCPcounter” package (Becht et al., 2016). MCPcounter is a method for quantifying the relative abundance of immune cells in different tissues using optimized marker genes. Ten different cell types including T cells, B lineage, cytotoxic T cells, NK cells, myeloid dendritic cells, monocytic lineage, CD8 cells, neutrophils, fibroblasts, and endothelial cells were assessed. Xcell is a tool that calculates independent enrichment scores of 64 kinds of immune and stromal cells (Aran et al., 2017). This algorithm was used to verify the findings from CIBERSORT and MCPcounter.

### Statistical analysis and additional bioinformatic

The correlation network involving different immune cell types was generated using R package “corrr”. The correlations between immune cells and genes were determined using Spearman correlation analysis, and the linear relationships between gene expression levels were evaluated by the Pearson correlation analysis. All statistical analyses were performed using the R software, and the comparison between different groups were done by the Wilcoxon test. All statistical tests were performed bilaterally, and *p*-values less than 0.05 were deemed to be statistically significant.

## Results

### Identification of DEGs and enrichment analysis

**Figure 1:**
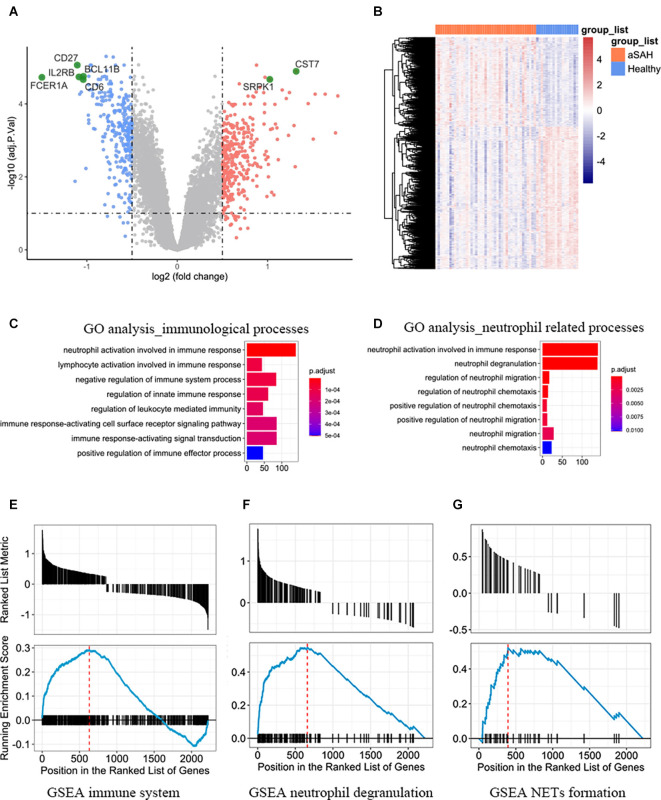
**(A)** Volcano plot and **(B)** heatmap showing the expression profiles between aSAH and control groups. **(C)** The top eight functions associated with immunological processes between aSAH and healthy samples. **(D)** The top eight functions associated with neutrophil. **(E–G)** Representative enriched pathways of GSEA. Each graph represents immune system, neutrophil degranulation, and neutrophil extracellular traps (NETs) formation.

After normalization and linear model fitting for gene expression in both groups, a total of 2,220 genes were identified as DEGs, with 856 upregulated and 1,364 downregulated genes, based on the adjusted p-value less than 0.05 and absolute log2(foldchange) more than 0.25. Volcano plot was then performed to present gene distribution (Figure 1A), and hierarchical clustering plots were used to check the discriminatory ability of DEGs in blood samples between aSAH and healthy controls (Figure 1B). The DEGs were further analyzed to explore the potential function and information of the genes in the progression of aSAH. The first 12 functions of biological process (BP) in the GO term were shown in an enrichment cluster diagram (Supplementary Figure 1). The results suggested the potential functions were mainly enriched in immunological process, especially related with neutrophil (including neutrophil activation and neutrophil degranulation). The top 10 functions associated with immunology and neutrophil were then extracted and presented as bubble plot, as shown in Figures 1C,D. As for GSEA, the results were shown in Figures 1E–G, which revealed that immune system, neutrophil degranulation, and neutrophil extracellular traps (NETs) formation were enriched in aSAH group compared with healthy controls.

**Figure 2:**
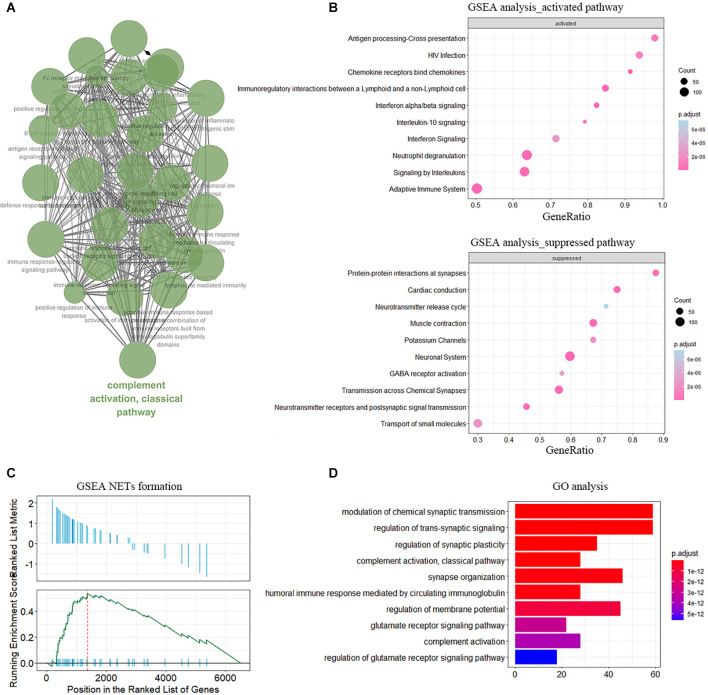
Functional enrichment of DEGs in sample of arterial samples dataset. **(A)** The interaction networks of enriched biological processes analyzed by ClueGO. **(B)** GSEA showing the enriched pathway in neutrophile degranulation. **(C)** Enrichment of NETs formation term analyzed by GSEA. **(D)** GO enrichment analysis in the aSAH blood samples.

Then we obtained DEGs between IA walls from aSAH and healthy arterial tissues based on RNA-seq data. The genes were then input to the Cytoscape to visualize the interaction network of biological process. The results were shown in Figure 2A. GO analysis and GSEA analysis further validated the current findings, which suggested immunity is involved in the aSAH process (Figures 2B–D). Enrichment of NETs formation was also found from GSEA (Figure 2C).

### Immune landscape in the peripheral blood between aSAH and control samples

**Figure 3:**
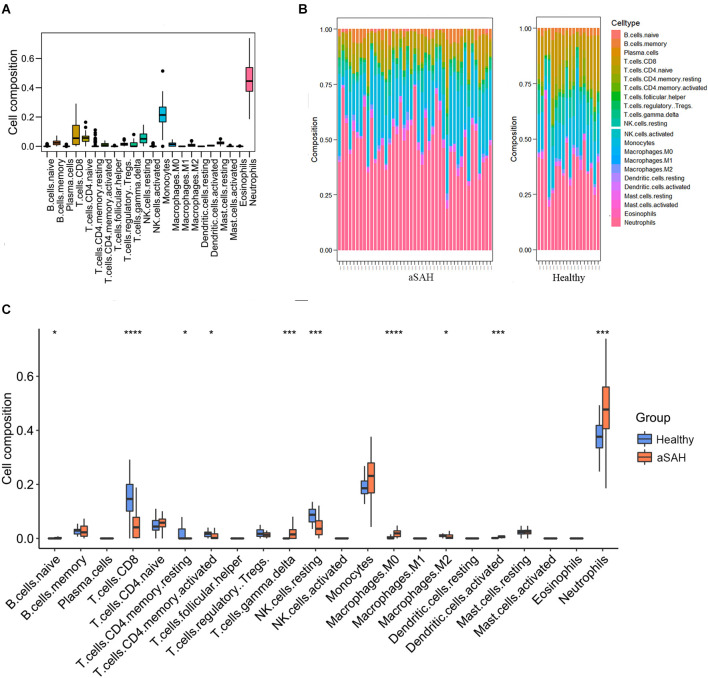
Immune landscape in the arterial samples between aSAH and healthy groups analyzed by CIBERSORT method. **(A)** The heatmap showing the distribution of the 22 immune cells. **(B)** The bar chart showing the enrichment scores in aSAH and healthy control groups. **(C)** The boxplot comparing the cell compositions between aSAH and control groups. The statistical difference was compared by the Wilcoxon test (*p < 0.05, ***p < 0.001, ****p < 0.0001).

Enrichment analysis showed that immune-related functions and signaling pathways were predominantly activated in aSAH blood samples compared to healthy controls. In order to further investigate the different immune landscapes in peripheral blood samples between aSAH patients and healthy persons. We analyzed the proportion and expression level of different immune cells in each sample using the CIBERSORT method. Figure 3A clusters the distribution of the 22 immune cells. Most immune cells were rarely expressed in blood samples, while neutrophils were dominant in blood samples (Figure 3B). The immune landscape results suggested that neutrophils were abnormally upregulated compared with those in the control group (Figure 3C). Besides, the peripheral blood samples in the aSAH group had significantly increased M0 macrophages scores. In contrast, the scores for lymphocyte in the peripheral blood of aSAH patients were significantly decreased than those of the control participants, such as CD8 T cells, memory CD4 cells, and NK cell.

### Predictive model construction and evaluation of the key genes

**Figure 4:**
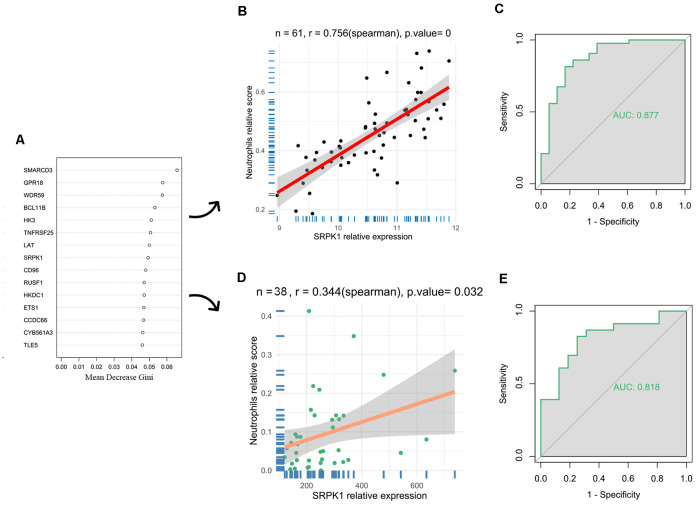
Identification of key genes. **(A)** Results of random forest showing the top 15 genes. To select key genes correlated with above infiltrated immune situation. **(B)** The correlation coefficient between SRPK1 and neutrophils was 0.76 (*p* < 0.001) in the blood sample dataset. **(C)** The area under the curve (AUC) was 0.877 (95% CI: 0.773–0.982). **(D)** The correlation coefficient between SRPK1 and neutrophils was 0.34 (*p* = 0.032) in the arterial sample dataset. **(E)** The area under the curve (AUC) was 0.818 (95% CI: 0.681–0.954).

To further investigate the genes mostly associated with the inflammatory reactions in the progression of aSAH, we performed random forest to select key genes correlated with above infiltrated immune situation (Figure 4A). In addition, recent publications on the same field were reviewed to identify candidate genes. For the concerned genes, we performed ROC and calculated the AUC values to evaluate the accuracy of the models. As a result, SRPK1 was selected as a potential gene for further exploration. The AUC of the model was 0.88, with 95% confidence intervals ranging from 0.77 to 0.98, demonstrating the robustness of SRPK1 in distinguishing between aSAH and healthy blood samples (Figures 4B,C). Similarly, the AUC was 0.82, with 95% confidence intervals ranging from 0.68 to 0.95 in the model of tissue samples (Figures 4D,E).

To explore the downstream biological processes in which SRPK1 may be involved, we obtained DEGs between the SRPK1 high expression group and low expression group in the blood samples dataset (Supplementary Figure 2). Then we performed GO analysis to predict the underlying biological functions of SRPK1 relevant to immunological process (Supplementary Figure 3) and neutrophil related process (Supplementary Figure 4). These results all suggested that high expression of SRPK1 was associated with neutrophil activation and neutrophil degranulation.

### Relationships between SRPK1 and immune cells and gene set variation analysis

**Figure 5:**
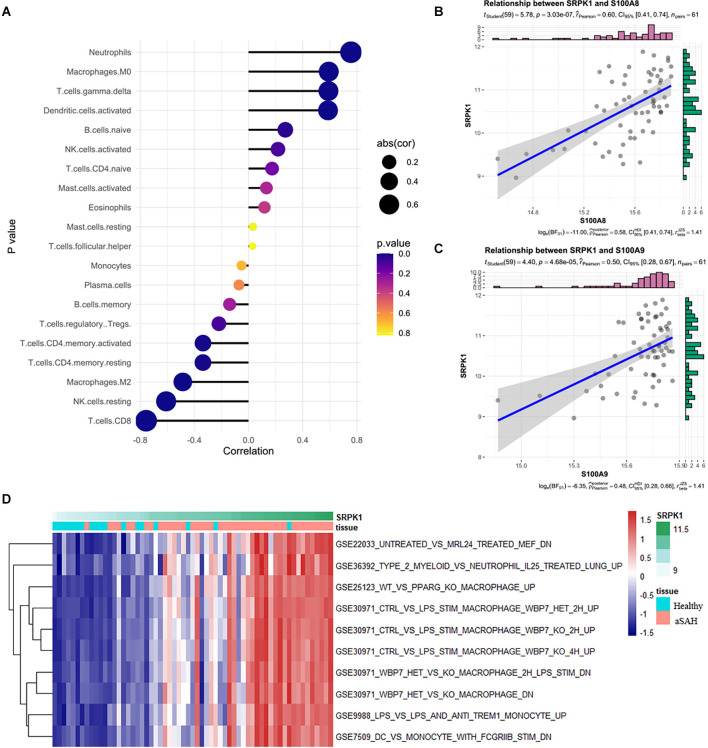
**(A)** Relationships among infiltration levels of 22 immune cell types and SRPK1 expression profiles by Spearman’s analysis. **(B,C)** Correlation of marker gene of neutrophils S100A8 and S100A9 with SRPK1. The correlation coefficient was 0.60 (*p* < 0.001) and 0.50 (*p* < 0.001) respectively. **(D)** GSVA of significant immunologic signature pathways between aSAH and healthy samples along with SRPK1 expression in the blood sample dataset.

Next, we analyzed cell infiltration data and expression data to explore the correlation between SRPK1 and 22 types of immune cells. As presented in Figure 5A, SRPK1 was significantly associated with neutrophils among all the immune cells. It also showed strong correlations with S100A8 and S100A9, the marked genes of neutrophils (Figures 5B,C). GSVA showed that along with the upregulation of SRPK1 expression, inflammation-related pathways also showed excessive activation (Figure 5D).

### Expression profile of transcription factor ZNF281 and its structure

**Figure 6:**
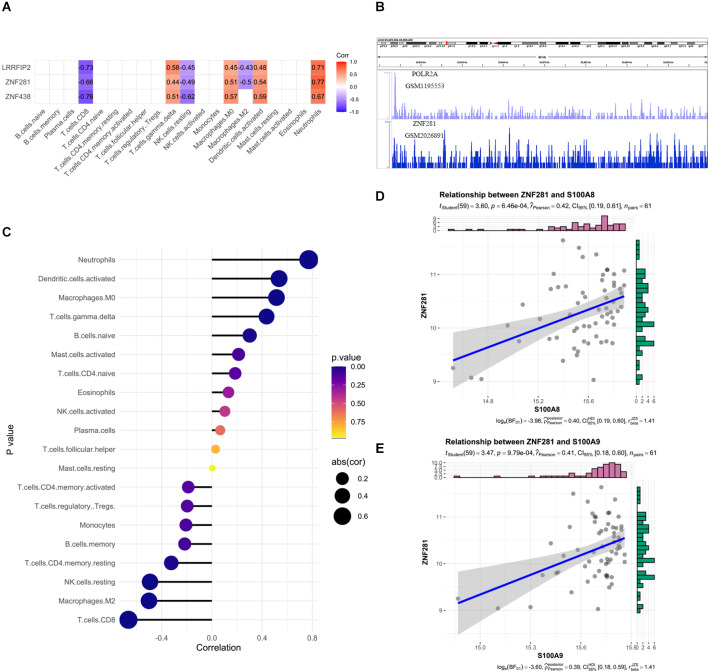
**(A)** The correlation heatmap visualized the relationship between transcription factor (TF) which may regulate SRPK1 expression and immune cells. **(B)** The loci of ZNF281 and POLR2A at chromosome 6 indicates ZNF281 binds to the promoter of SRPK1. **(C)** Relationships among infiltration levels of 22 immune cell types and ZNF281 expression profiles by Spearman’s analysis. **(D,E)** Correlation of marker gene of neutrophils: S100A8 and S100A9 with ZNF281. The correlation coefficient was 0.42 (*p* < 0.001) and 0.41 (*p* < 0.001) respectively.

Next, we predicted transcription factor (TF) which may regulate SRPK1 expression (Figure 6A), and selected ZNF281 as potential TF due to its high correlation (Figure 6B). We assumed that ZNF281 can bind with polymerase (RNA) II (DNA directed) polypeptide A (POLR2A), which could regulate synthesis of message RNA. Then we adopted similar strategy in ZNF281 and discovered that high ZNF281 expression associated with neutrophils scores (Figure 6C). The RNA-seq data verified our findings (Figures 6D,E).

**Figure 7:**
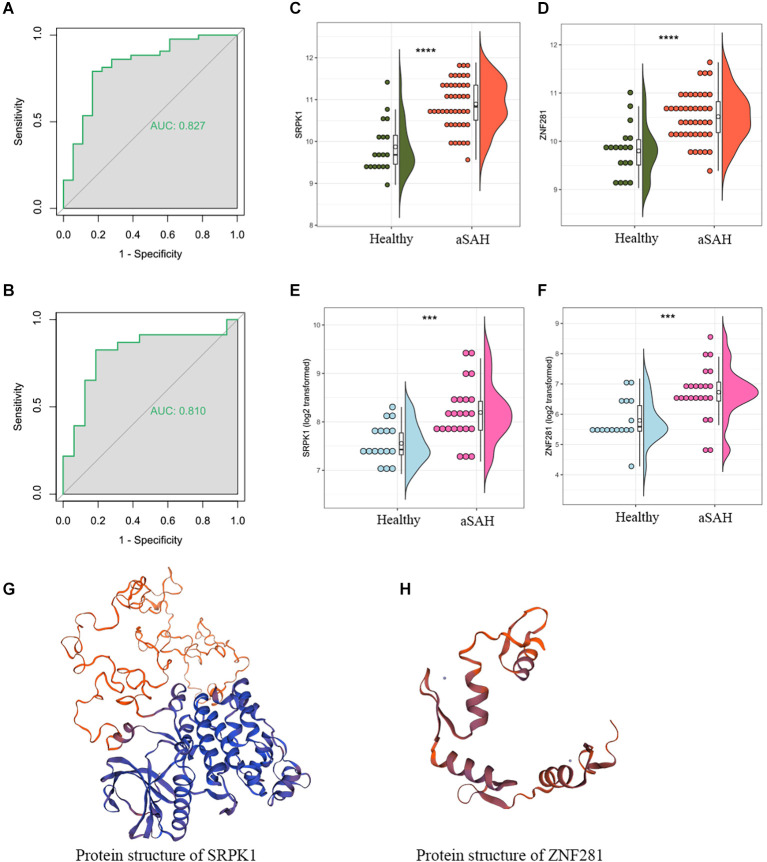
The area under the curve (AUC) of ZNF281 was 0.877 and 0.810 in the **(A)** blood sample and **(B)** tissue sample dataset, respectively. **(C,D)** Expression profile of SRPK1 and ZNF281 in the blood sample dataset and **(E,F)** in the tissue sample dataset. **(G,H)** Protein structures of SRPK1 and ZNF281 (***p < 0.001, ****p < 0.0001).

Tissue data was used to validate the predictive capability of ZNF281 (Figures 7A,B). The expression profiles of them were shown in Figures 7C–F. The results remained consistent in the blood sample as well as in the tissue sample. We also presented the protein structures of SRPK1 and ZNF281 in Figures 7G,H.

**Figure 8:**
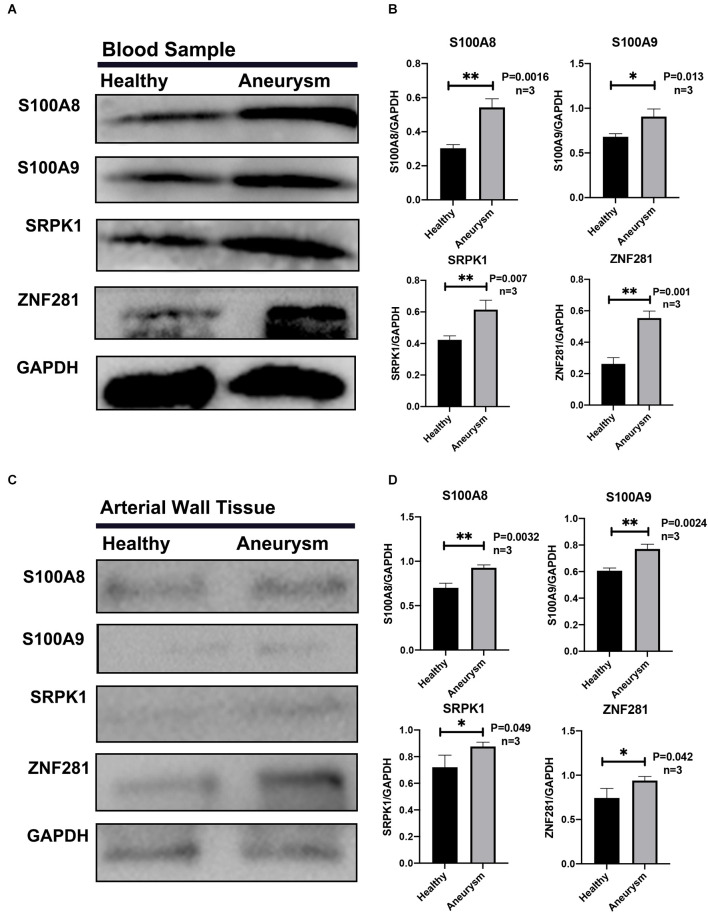
**(A)** Representative Western blot bands of S100A8, S100A9, SRPK1, and ZNF281 expression in blood samples in different groups. **(B)** Quantitative analysis of S100A8, S100A9, SRPK1, and ZNF281 expression in blood samples in different groups. *n* = 3/group. **(C)** Representative Western blot bands of S100A8, S100A9, SRPK1, and ZNF281 expression in arterial walls and aneurysms walls from different groups. **(D)** Quantitative analysis of S100A8, S100A9, SRPK1, and ZNF281 expression in arterial samples in different groups. *n* = 3/group. Healthy control: Healthy middle meningeal arteries; Aneurysm: Ruptured aneurysm tissue (^*^p < 0.05, ^**^p < 0.01).

**Figure 9:**
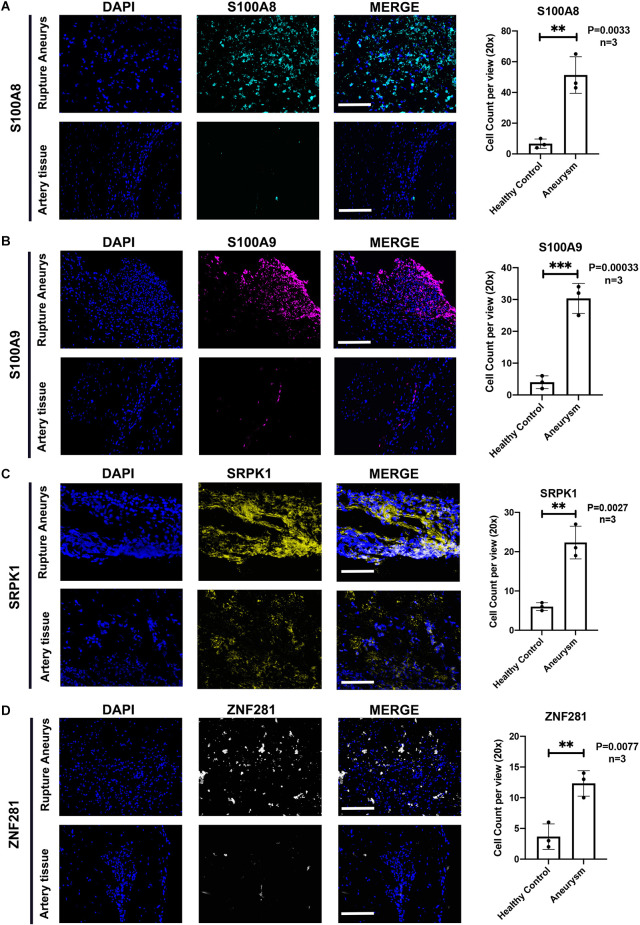
**(A)** Representative photographs of immunofluorescence staining showing S100A8 (marked with green) and DAPI (marked with blue) in healthy group and aSAH group, and quantitative analysis of positive cells. *n* = 3/group; *p* = 0.0033. **(B)** Representative photographs of immunofluorescence staining showing S100A9 (marked with red) and DAPI (marked with blue) in healthy group and aSAH group, and quantitative analysis of positive cells. *n* = 3/group; *p* = 0.0003. **(C)** Representative photographs of immunofluorescence staining showing SRPK1 (marked with yellow) and DAPI (marked with blue) in healthy group and aSAH group, and quantitative analysis of positive cells. *n* = 3/group; *p* = 0.0027.** (D)** Representative photographs of immunofluorescence staining showing ZNF281 (marked with yellow) and DAPI (marked with blue) in healthy group and aSAH group, and quantitative analysis of positive cells. *n* = 3/group; *p* = 0.0077. Healthy control: Healthy Middle meningeal arteries; Aneurysm: Ruptured aneurysm tissue. Scale bar: 75 μm (^**^p < 0.01, ^***^p < 0.001).

The Western-blotting assay was performed to detect expression level of neutrophil related proteins. We observed that both S100A8 and S100A9 were elevated in the aSAH group both in the blood samples and arterial tissues (Figures 8A–D). The expression profiles of these key genes were also examined by immunofluorescence assay. The results suggest that arterial tissues in the aSAH group tends to accumulate more SRPK1, ZNF281, S100A8, and S100A9 in cytoplasm (Figures 9A–D).

### MCPcounter and xcell verification

**Figure 10:**
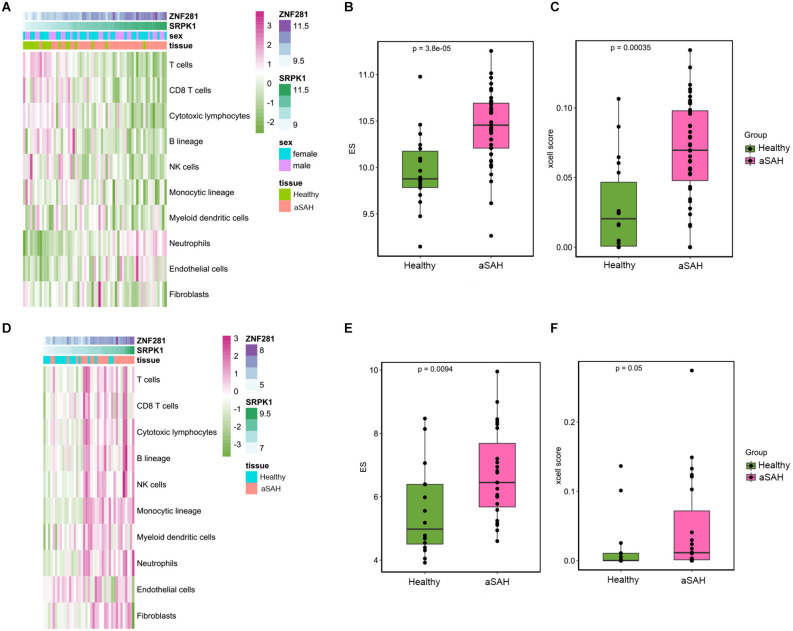
Immune landscape in the tissue samples between aSAH and healthy samples analyzed by MCPcounter and xcell algorithm. **(A)** The heatmap showing the overall enrichment of 10 immune cells in the blood sample. **(B)** The boxplot showing the MCPcounter enrichment scores of neutrophils between aSAH and healthy groups. **(C)** The boxplot showing the xcell enrichment scores of neutrophils between aSAH and healthy groups. **(D)** The heatmap showing the MCPcounter results of the arterial sample dataset. **(E,F)** The boxplot showing the MCPcounter and xcell enrichment scores of neutrophils between aSAH and healthy groups in the arterial sample dataset.

MCPcounter was a different algorithm to calculate the landscape of immune cell infiltration, and was used to verify the present findings from different perspectives. The abundance scores of the 10 subpopulations of immune cells infiltrating the aSAH and healthy blood samples were compared and were shown in the heatmap (Figure 10A). Consistent with CIBERSORT, we found that the infiltration of neutrophils was significantly higher in aSAH patients compared to control ones. SRPK1 and ZNF281 also showed significant relationship with neutrophil scores based on this algorithm, as the expression of SRPK1 increased, neutrophil enrichment also became greater (Figures 10B,C). Similar findings were presented in Figures 10D–F using the RNAseq dataset.

**Figure 11:**
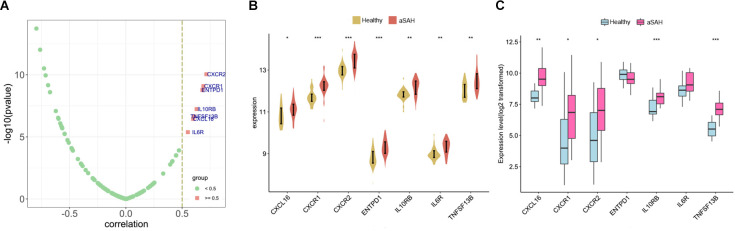
**(A)** Correlation of immunomodulators with SRPK1. **(B,C)** The expression difference of immunomodulators with correlation coefficient more than 0.5 between aSAH and control tissues in the blood and tissue samples (*p < 0.05, **p < 0.01, ***p < 0.001).

Next, various immunomodulators were employed to detect the correlation with SRPK1 (Figure 11A). Our analysis demonstrated that the top seven immunomodulatory factors that positively associated with the expression levels of SRPK1 were CXCR2, CXCR1, ENTPD1, IL10RB, TNFSF13B, CXCL16, and IL6R. Meanwhile, these factors were highly expressed in blood samples and tissue samples from aSAH patients (Figures 11B,C).

## Discussion

Although significant progress has been made in terms of medical care and surgical treatment, aSAH remains a destructive disorder with high morbidity and mortality. For a long time, immune cell infiltration has been shown to have a major role in the onset and progression of aSAH. This complex process not only triggers neuroinflammation in the brain, but also contributes to a systemic inflammatory response (Lee et al., 2012). The excessive inflammatory response and subsequent tissue damage is mediated by the infiltration and activation of various immune cells from brain tissue and blood sources, which ultimately lead to brain injury (Savarraj et al., 2018; Coulibaly and Provencio, 2020). The engagement of the inflammatory reaction in the process resulting in rupture is also supported by observational studies with human patients. For example, consumption of medications with anti-inflammatory effects, such as statins and NSAIDs, may reduce the risk of SAH due to IA rupture (Zeyu et al., 2021). Therefore, it is crucial to thoroughly explore the role of infiltrating immune cells in SAH. In this study, we assessed the difference in immune infiltration profile between aSAH and healthy arterial samples using three algorithms including CIBERSORT, MCPcounter, and xcell. Different algorithms use different statistical methods for deconvolution analysis, for example, CIBERSORT is based on linear support vector regression (Newman et al., 2015). Similar results were obtained by different algorithms, which confirmed the reliability of the findings. Eventually we identified SRPK1 as a potential diagnostic biomarker and possibly a potential therapeutic target for aSAH through extensive bioinformatics analysis.

Although much is known about inflammation and the progression of aSAH, previous studies have mainly focused on monocytes/macrophages (Thomas et al., 2018). The potential role of neutrophils in promoting the progression of aSAH remains insufficiently described. In this study, GO enrichment analysis showed that DEGs with higher expression in the whole blood from aSAH patients were related to immune process, especially pathways related to neutrophil-associated response. Further enrichment analysis of tissue samples validated our findings. Clinical observations suggest that neutrophils may serve as a potential contributor in the development of IAs. On the one hand, circulating neutrophils carry IA associated features and are potential molecular biomarker to distinguish patients with IA (Tutino et al., 2018b). On the other hand, neutrophils promote the rupture of IAs and elevated peripheral neutrophils are significantly associated with prognosis of patients following subarachnoid hemorrhage (Zhang Y. et al., 2021). Experimental studies have also demonstrated the role of neutrophils in the pathophysiology of IAs. For example, a higher degree of neutrophil infiltration was observed in ruptured IAs than unruptured ones (Tulamo et al., 2006). It has been noted that neutrophils may play a key role in the maintenance and aggravation of the inflammatory response (Miyata et al., 2019; Kushamae et al., 2020). In response to the cytokines present *in situ*, neutrophils can generate a substantial amount of pro-inflammatory factors, such as TNF-α and PGE2 (Kang et al., 2020; Kushamae et al., 2020). This process may act in conjunction with macrophages activated by the cytokines produced by neutrophils to exacerbate the inflammatory response.

Recent studies suggest that NETs are involved in the progression of intracranial aneurysm (Kang et al., 2020; Korai et al., 2021). The neutrophils infiltrate into the damaged brain tissue following SAH. Upon stimulation by various biochemical factors, neutrophils release DNA, granulins, and histones. These fibrous matrices are known as NETs. Formation of NETs promote tissue-damaging immunopathology in an inflammatory state. There is growing evidence that NETs exacerbate inflammatory events after SAH and impair revascularization and increase post-stroke blood-brain barrier damage. Furthermore, resolution of formed NETs or pharmacological removal of NETs by inhibition of peptidylarginine deiminase has shown to be a potential therapeutic strategy to prevent IA rupture (Zeng et al., 2022).

NET generation is a physiologic way of cell death called NETosis (Kolaczkowska and Kubes, 2013). Components of NETs include DNA fragments, histones, and neutrophil granule proteins. The family members of S100 proteins S100A8 and S100A9 originate predominantly from neutrophils and monocytes which are involved in neutrophil activation and NET-induced inflammation (Austermann et al., 2018). Increasing evidence suggests a crucial role for the S100 family proteins in driving inflammation in cancers and in non-cancer diseases. For example, NET-S100A9-MMP9 acts as a critical component in connective tissue destruction (Akiyama et al., 2019). Levels of NET protein and S100A8 have also been shown to be important markers for predicting the prognosis of patients with ovarian cancer (Muqaku et al., 2020).

SRPK1 is a highly conserved protein in eukaryotic organisms which belongs to the SRPK family and contained a protein kinase-like domain. It has been found to be expressed in both the cytoplasm and nucleus. Previous studies reported that SRPK1 was involved in the regulation of a number of cellular processes, such as chromatin structure, mRNA maturation, and reproductive cell development (Giannakouros et al., 2011). By regulating the phosphorylation of SR splicing factors, SRPK1 can influence the splicing of pre-mRNAs and thus gene expression. Reports have shown that elevated levels of SRPK1 expression are associated with risk and prognosis in liver, breast, and lung tumors, while the specific mechanisms of SRPK1 in cancer progression remain to be classified (Nikas et al., 2019). In addition, previous studies have revealed that SRPK1 is expressed in the human central nervous system (Mytilinaios et al., 2012). It may have a key role in regulating the expression of neuron-specific protein isoforms, including the expression of various neurotransmitter receptor subtypes. SRPK1 has recently been suggested as a potential new molecular target that could be employed to facilitate the treatment of patients with early-stage gliomas, owing to its ability to regulate cell growth, metastasis, chemosensitivity and glioma angiogenesis (Sigala et al., 2016).

Also, researchers have found that silencing of SRPK1 increases vascular smooth muscle cells proliferation and promotes vascular remodeling in IA, suggesting SRPK1 as a molecular target for treatment of IA (Li and Wang, 2019). In the present study, we assumed that SRPK1 exerts a facilitative effect on the progression and rupture of IAs, and this process was probably regulated by ZNF281.

We also observed the dysregulation of inflammation and a potential role of immunomodulators in our bioinformatics analyses. CXCR1 and CXCR2 are receptors for IL8 which is a powerful neutrophil chemotactic factor (Ludwig et al., 1997; Osuka et al., 2021). Activation of CXCR1 and CXCR2 not only recruits neutrophils to the lesion, but also triggers extrusion of NETs (Teijeira et al., 2020). TNFSF13B is also known as a marked gene of neutrophil activation (Besteman et al., 2020). CXCL16 was reported to have great value for predicting UIA rupture, and is significantly associated with poor outcome in patients with aSAH (Shan et al., 2021; Xu et al., 2021). It is possible that SRPK1 promotes SAH progression by interacting with these immunomodulators, for example by affecting the maturation of mRNAs transcribed by target genes. Future studies are needed to investigate the underlying mechanisms.

Several limitations of the present study need to be resolved by further research. First, survival data were not available, it was therefore unable to explore the association between the key genes and prognosis of patients. Second, additional experiments to investigate the specific function of the concerned genes are required. Although we uncovered the predictive value of SRPK1 in aSAH diagnosis; further *in vivo* and *in vitro* studies should concentrate on the signaling pathways involved in the interaction of SRPK1 with immune factors in aSAH. For example, whether SRPK1 promotes aSAH progression by interacting with CXCR1 and CXCR2.

In summary, the immune landscape of aSAH was comprehensively stratified and quantified. Neutrophils represent an important immune cell population involved in the pathophysiological progression following aSAH. The key genes SRPK1 and ZNF281 could serve as effective biomarkers to distinguish patients with aSAH, and potential therapeutic targets. Moreover, several immunomodulators such as CXCR1 and CXCR2 also appear to be involved in the progression of SAH. Our findings present convincing insights into the pathogenesis of and potential therapeutic targets for SAH and provide a foundation for future studies. The findings of the present study should be validated in a larger cohort.

## Data Availability Statement

The datasets presented in this study can be found in online repositories. The names of the repository/repositories and accession number(s) can be found in the article/Supplementary material.

## Ethics Statement

Ethical review and approval was not required for the study on human participants in accordance with the local legislation and institutional requirements. Written informed consent for participation was not required for this study in accordance with the national legislation and the institutional requirements.

## Author Contributions

XW: drafting/revision of the manuscript for content, acquisition of data, study concept or design, analysis or interpretation of data. DW: drafting/revision of the manuscript for content, analysis or interpretation of data. CY: major role in the acquisition of data, study concept or design. LM: major role in the acquisition of data, study concept or design. All authors contributed to the article and approved the submitted version.

## Funding

This research was funded by the National key Research & Development Program of China, grant number 2018YFA0108604 & 2018YFA0108603; Clinical Incubation Program of West China Hospital, grant number 2018HXFH008; and Science and Technology Department of Sichuan Province, grant number 2020YFQ0009. The funders had no role in the design or conduct of this research.

## Conflict of Interest

The authors declare that the research was conducted in the absence of any commercial or financial relationships that could be construed as a potential conflict of interest.

## Publisher’s Note

All claims expressed in this article are solely those of the authors and do not necessarily represent those of their affiliated organizations, or those of the publisher, the editors and the reviewers. Any product that may be evaluated in this article, or claim that may be made by its manufacturer, is not guaranteed or endorsed by the publisher.
