# Supplementary material for: Identification of the key immune-related genes in aneurysmal subarachnoid hemorrhage
Source: Front Mol Neurosci. 2022 Sep 12;15:931753. doi: 10.3389/fnmol.2022.931753 (PMC9511034; doi:10.3389/fnmol.2022.931753)
Supplement: Supplementary file 1 [file Data_Sheet_1.docx]

Supplementary Material


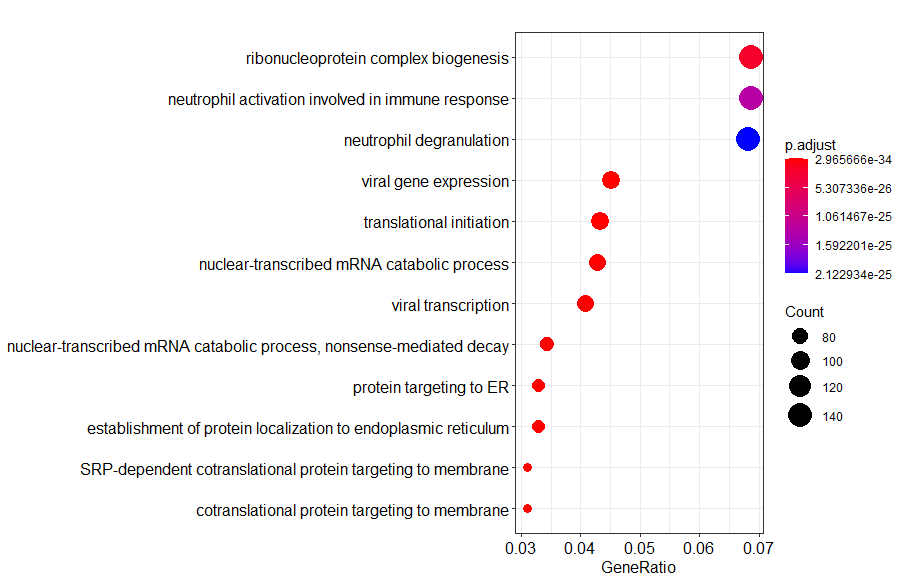


**Supplementary Figure 1.** The top 12 GO terms of aSAH and healthy control samples in the GSE36791 dataset.

**
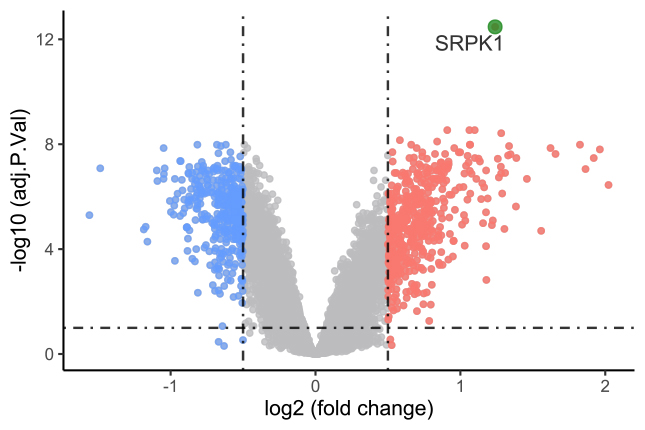
**

**Supplementary Figure 2.** DEGs identified in the GSE36791 dataset according to SRPK1 expression. A total of 2660 genes are obtained.


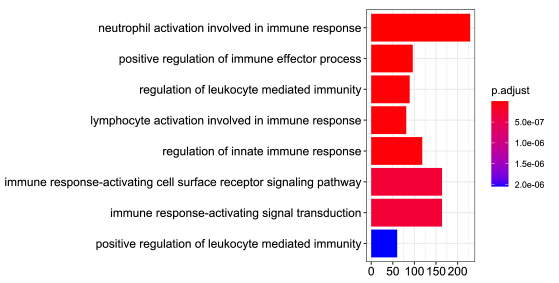


**Supplementary Figure 3.** GO analysis of DEGs in immune-related enrichment terms.


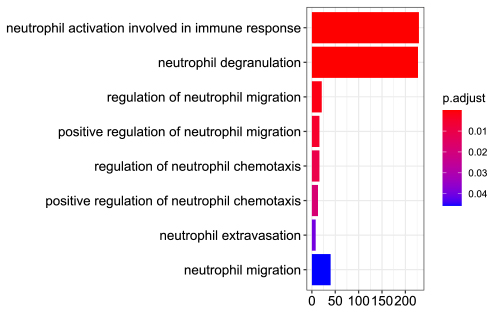


**Supplementary Figure 4.** GO analysis of DEGs in neutrophil-related enrichment terms.
